# Clavicle fracture and triathlon performance: a case report

**DOI:** 10.1186/s13256-024-04482-7

**Published:** 2024-04-03

**Authors:** Lennart Gerdesmeyer, Rainer Burgkart, Amol Saxena

**Affiliations:** 1 Department of Orthopaedics and Sports Orthopaedics, Klinikum Rechts der Isar, Technical University of Munich, Ismaninger Str. 22, 81675 Munich, Germany; 2 PAMF-Sutter Department of Sports Medicine, 795 El Camino Real, Clark Building, Level 3, Palo Alto, CA 94301 USA

**Keywords:** Return to sports, Accelerated healing, Electromagnetic field therapy, Fracture healing, Treatment guidelines, Collarbone fracture, Case report

## Abstract

**Background:**

Collarbone fracture is a common injury, particularly among athletes involved in contact sports and participating in endurance activities. Conventional treatment requires surgery and postoperative immobilization, resulting in an average return-to-sport timeframe of approximately 13 weeks. This case challenges the established treatment protocols, aiming to expedite recovery and enable a quicker resumption of high-intensity athletic activities.

**Case presentation:**

A 24-year-old Caucasian athlete completed a Half-Ironman Triathlon (70.3) merely three weeks post-collarbone fracture. Utilizing Extracorporeal Magneto-Transduction Therapy (EMTT) alongside surgical intervention, the patient achieved accelerated healing and remarkable performance outcomes without encountering any adverse effects.

**Conclusions:**

The integration of EMTT into the treatment paradigm for bone fractures alters the traditional understanding of recovery timelines and rehabilitation strategies. This case highlights the potential benefits of electromagnetic wave therapy in expediting the healing process and enabling athletes to resume high-level sports activities at an earlier stage.

## Introduction

Clavicle fractures account for approximately 10% of all sport-related fractures [1, 2]. Additionally, they represent 2.6% of all fractures and 44% of fractures occurring in the shoulder region [3]. Allman classified clavicle fractures into three types based on the location of the fracture: Type 1 involves the middle third of the clavicle, Type 2 affects the distal end, and Type 3 affects the medial third [4]. Neer further subdivided lateral clavicle fractures into five subtypes based on the status of the coracoclavicular ligaments [5]. In our patient's case, he suffered from a Type 2a fracture, indicating that the ligaments located laterally were not affected. The optimal treatment protocol for clavicle fractures remains a topic of debate. Therefore, managing these fractures depends on the hospital´s and physicians’ preferences. Evidence proposes a higher risk of developing non-union with non-surgical treatment [6, 7]. Given the significant displacement of the medial fragment in our patient's case, we concluded that surgery may lead to a better outcome compared to conservative therapy. According to the evidence-based clinical practice guidelines from the AAOS (2022), we recommended immobilization with a sling for a few days after surgery. The surgical treatment for clavicular fractures is becoming increasingly common. In Finland, the incidence of surgical treatment significantly increased from 1.3 per 100,000 persons in 1987 to 10.8 per 100,000 persons in 2010 [8]. The average time off work is 49 days for operative treatment and 47 days for non-operative treatment [9]. The average time required to return to sports (including various surgical and non-surgical treatments) is approximately 13.7 weeks. Furthermore, only 81% of patients can return to their pre-injury level [10]. Considering both personal factors and the healthcare costs associated with a prolonged absence, there is an urgent need to investigate new rehabilitation protocols to expedite the return to daily life and sports activities. In our case, the patient used Extracorporeal Magneto-Transduction Therapy (EMTT) to recover faster.

According to some older reports, electrical stimulation has been used in medical treatment since 1841 [11]. One specific type of electromagnetic wave therapy is PEMF (pulsed electromagnetic field) therapy, which gained approval from the Food and Drug Administration (FDA) in 1979. PEMF devices have been used as a modality for treating various osteogenic disorders, including fracture healing [12–14] or reducing bone loss associated with osteoporosis [15, 16]. EMTT, on the other hand, is a newer device that falls within the category of electromagnetic treatment. It differs from PEMF in a few physical aspects. EMTT utilizes high-intensity PEMFs with a magnetic field strength of up to 150 mT. It operates at an effective transduction power exceeding 60 kT/s and has an oscillating frequency ranging between 100–300 kHz. These specifications enable EMTT to penetrate tissues up to 18 cm deep. In comparison, PEMF devices typically have lower power outputs, falling below 60 kT/s [17, 18].

## Case presentation

**Fig. 1:**
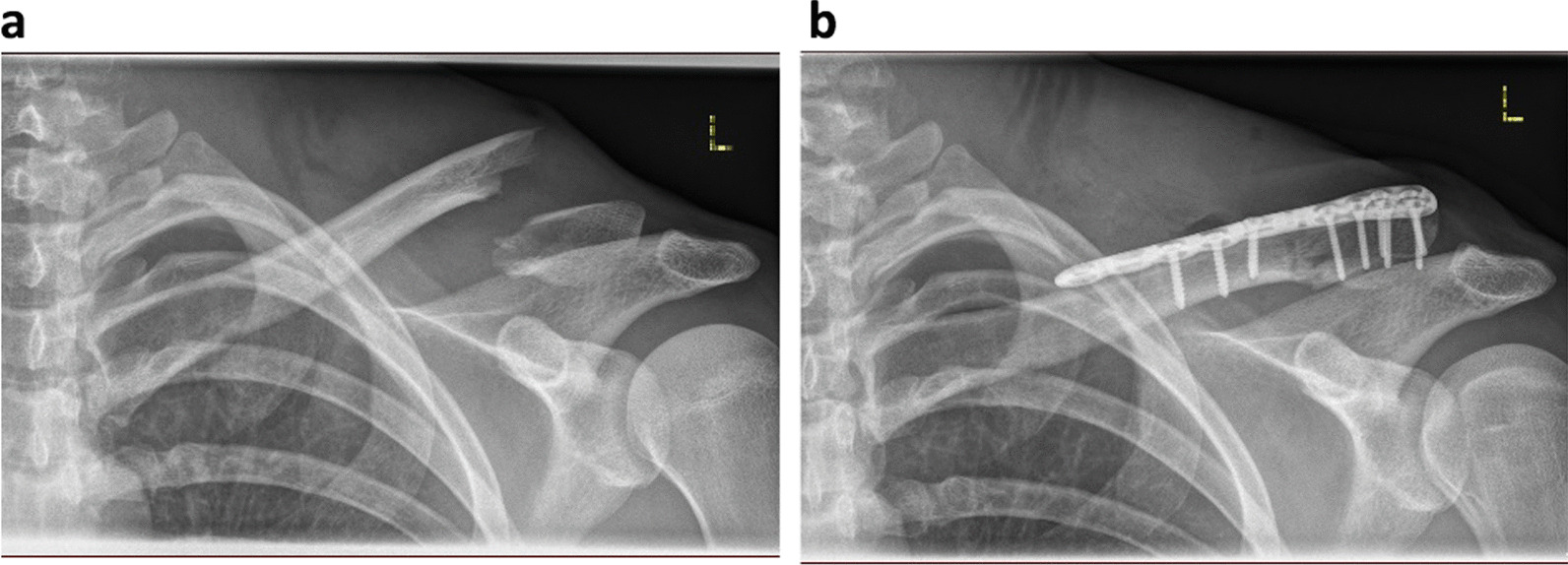
**a** Radiograph showing left clavicle fracture Typ IIa after the bike accident. **b** Post-surgery radiograph showing left clavicle plate system (VariAx) after plate osteosynthesis

We present the case of a 24-year-old Caucasian non-smoking male patient training for mid and long-distance triathlon competitions. While road cycling, he had an accidental fall and suffered a fracture in his left lateral clavicle (Fig. 1a). Based on the Neer classification, the fracture can be categorized as a Type IIa fracture, which is located medial to the coracoclavicular ligament. There is noticeable displacement of the medial fragment, but the conoid and trapezoid ligaments remained intact. The patient underwent surgery the following day, during which the dislocated bone fragments were stabilized using a clavicle plate system (VariAx, Fig. 1b).

**Fig. 2:**
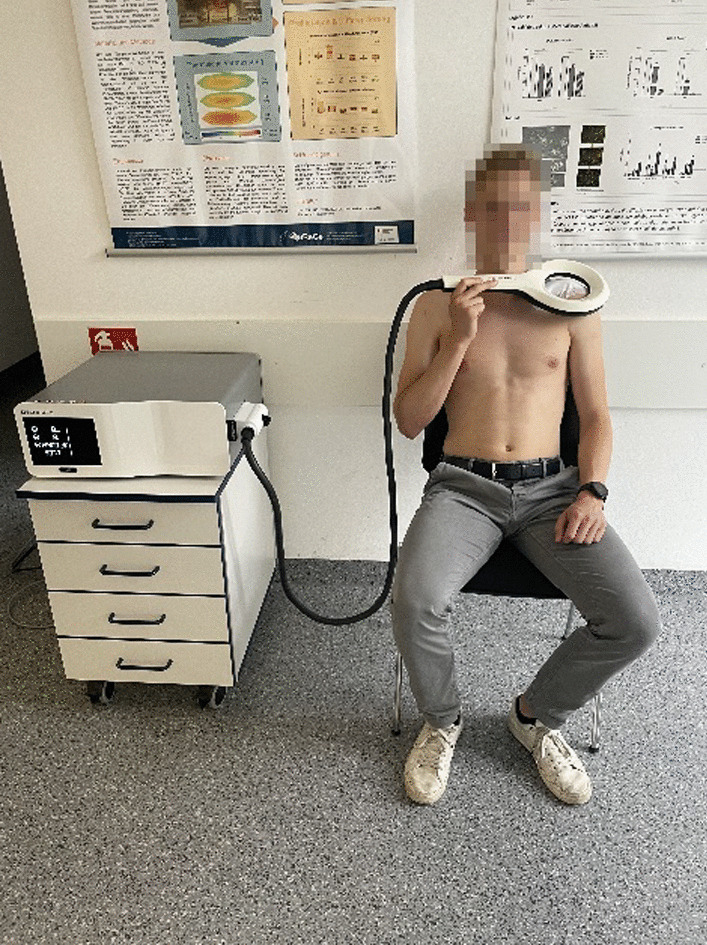
The patient applying extracorporeal magneto-transduction therapy (EMTT) 3 days post-surgery. Following protocol was used: Frequency 8 Hz, duration 30 min (14,400 impulses), and strength level 8 out of 8 (Device: STORZ Medical Magnetolith)

One day after surgery, he was discharged. Since the patient's goal was to participate in the upcoming Half-Ironman (consisting of a 1.2 mile/1.9 km swim, a 56 mile/90 km bike ride, and a 13.1 mile/21.1 km run) in three weeks, we immediately initiated EMTT stimulation on the same day (Table 1). We opted for a relatively intense treatment protocol using the Magnetolith device (STORZ Medical), stimulating for 30 min at a frequency of 8 Hz and magnetic field strength of approximately 80 mT (14,400 impulses per session). The patient does not experience any additional pain or discomfort during the application of EMTT. For the first two weeks, we repeated this protocol every other day (Fig. 2).

**Table 1:** Different applied therapy modalities

	Week 1	Week 2	Week 3
EMTT Stimulation	30 min, lvl 8, 8 Hz, every other day (− > 14.400 impulses)	30 min, lvl 8, 8 Hz, every other day (− > 14.400 impulses)	
Physiotherapy	Active movements limited to pain-free range (< 90° in every axis)	Active movements within a pain-free range (> 90° if painless)	Shoulder muscle strengthening with resistance bands
Sling (Gilchrist)	During daily life and activities	During daily life, omitted for sport activities	No need for the sling anymore
Range of motion (passive)	Flexion < 70° Abduction: < 60°	Flexion: < 100° Abduction: < 120°	Free range of motion
Range of motion (passive)	Flexion < 70° Abduction: < 60°	Flexion: < 100° Abduction: < 120°	Free range of motion

Physiotherapy exercises: 2–3 times a day for 15 min

min: minute; lvl: level; Hz: hertz

**Fig. 3:**
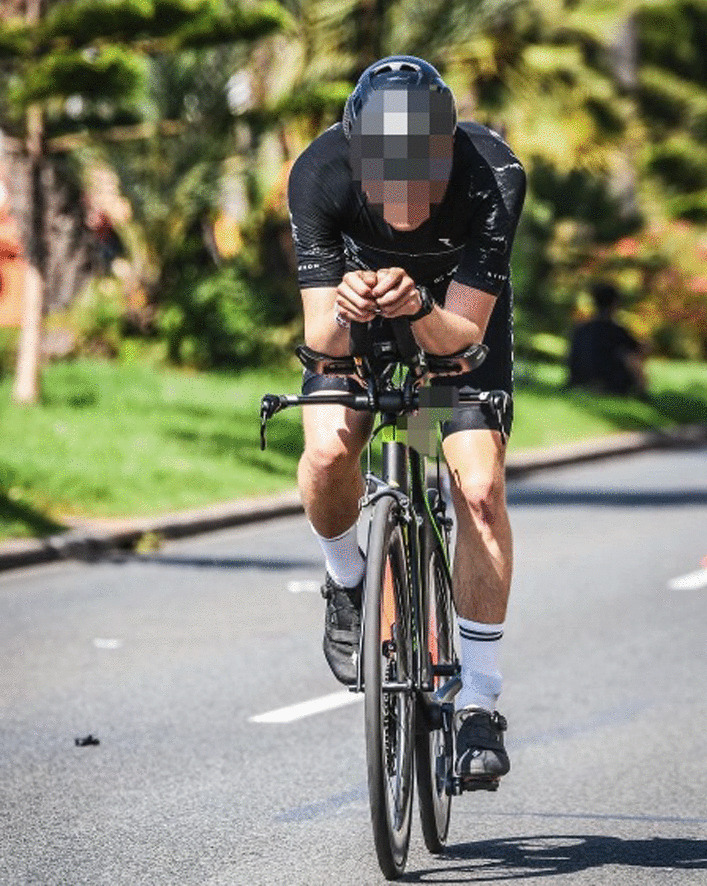
3 weeks post-surgery, during a triathlon race in a time trial (TT) position (flexion: 90°, adduction: 30°, internal rotation: 45°)

Additionally, we started with physiotherapeutic measures one day after the surgery (Table 1). In the first week, the patient's active and passive movements were limited to 90 degrees in flexion and abduction. Moreover, he resumed his training plan and began cycling on a home cycle trainer and running (Table 2), both while wearing a sling. The sling immobilization was maintained during the second week but omitted during the training sessions. Throughout the second week, the range of motion improved, allowing an elevation and abduction above 90° (Flexion: < 110°, Abduction: < 100°) under the guidance of the physiotherapist. Consequently, the athlete could perform bike training sessions on a TT-Bike (time trial) in conjunction with the TT-position (Fig. 3).

**Table 2:** Triathlon specific training performed by the patient

	Week 1	Week 2	Week 3
Running	Running with a sling (5 to 10 km/session)	Running at a moderate pace without a sling (10–20 km/session)	Intervals (including race pace)
Cycling	Indoor cycling using a smart trainer with Zwift (ca. 60 min/session)	Indoor cycling using a smart trainer with Zwift in a time trial (TT) position (60 to 150 min/session)	Outdoor cycling on a time trial (TT) bike (> 100 km/session)
Swimming			500 m, technique: Crawl

**Fig. 4:**
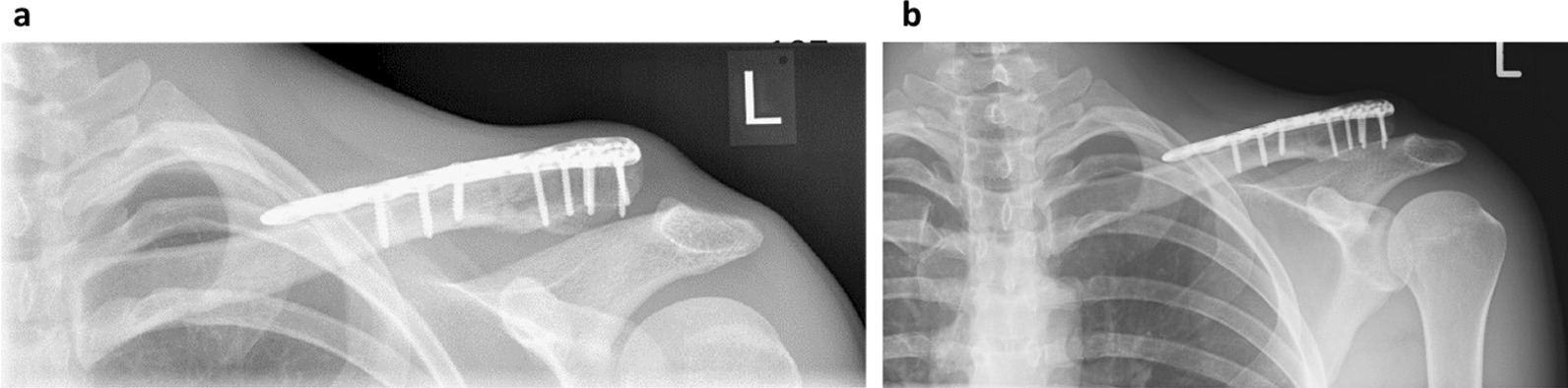
** a** Radiograph 6 weeks post-surgery. Condition after a lateral clavicle fracture treated with plate osteosynthesis and EMTT stimulation. The foreign material is intact, with no signs of loosening. The fracture gap appears partially healed. **b** Radiograph 10 weeks post-surgery showing complete bone healing following a clavicle fracture.

At the end of the second week, the athlete could run 20 km without experiencing any pain. Given the patient's report of being almost pain-free during daily activities (Table 3), we advanced to a more competition-oriented protocol, incorporating strength training using resistance bands. Furthermore, the athlete included brief high-intensity sessions into his exercise regimen. To test his ability to swim in the upcoming Half-Ironman, he swam the crawl for 500 m while wearing a wetsuit. To avoid causing pain, he refrained from pulling and pushing forcefully during the arm crawl phases. The Half-Ironman was completed three weeks after surgery, even achieving a personal best time. A cautious approach was taken in the swimming discipline, resulting in a more restrained swim performance. A clinical assessment after the competition did not reveal any deterioration in terms of painless range of motion. However, to ensure the correct positioning of the screws and plate, an X-ray evaluation was conducted six weeks after surgery (Fig. 4a). The fixation plate and all screws were intact and not loosened. The fracture gap already exhibited slight bony consolidation, indicating ongoing fracture healing. The treatment can be successfully completed (clinically) at this stage. To confirm complete bone healing radiologically too, the patient received one final follow-up X-ray image ten weeks post-surgery (Fig. 4b).

**Table 3:** Further assessment

	Week 1	Week 2	Week 3
Imaging	Pre- and post-surgery X-Ray		Post Race X-Ray
Pain (VAS score)	4–6	1–3	0–2

Pain management therapy was administered during the initial days following the surgery, involving the use of nonsteroidal anti-inflammatory drugs (NSAIDs) and ice application

- *First week:*
  o *Elbow, wrist, hand exercises*
  o *Pendulum, Circular (Codman's Exercises)*
  o *Isometric exercises*
  o *All movements were restricted to 90° (Flexion, Abduction)*
- *Second week:*
  o *Exercises from the first week*
  o *Arm lifting (Flexion) with support of the good arm (*< *90°, end of the week:* < *100°)*
  o *Walk Up Exercise against the wall (Active)*
  o *Passive movements supported by physiotherapist: Flexion:* < *110°, Abduction:* < *100°*
- *Third week:*
  o *Exercises from first and second week*
  o *Strengthening exercises with resistance band (pain adopted)*
  o *Passive movements supported by physiotherapist: Full range of motion (if painless)*

## Discussion

Numerous studies in the literature highlight the positive effects of PEMF therapy on various aspects of bone health, including chondrogenesis [19–21], cytoprotective effects against oxidative stress [22], anti-inflammatory effects [23–25], and bone metabolism. Studies conducted on osteoporosis-induced rats or mice (ovariectomized) have demonstrated improvements in bone mass, bone mineral density, and bone microarchitecture [15, 16]. Additionally, a wealth of research indicates accelerated fracture healing with PEMF therapy [12–14]. Potential mechanisms for these effects include increased osteoblast activity, enhanced osteoblast differentiation, inhibition of osteoclasts, and induction of angiogenesis [26–33].

The accelerated bone healing observed in our presented case aligns with current clinical research. A randomized, double-blind, sham-controlled pilot study involving 41 patients with radial fractures revealed significantly greater union extent and better functional outcomes in the group treated with electromagnetic stimulation compared to the control group [34]. Other clinical studies investigating the enhancement of fracture healing with PEMF have reported similar results [35–37]. However, there are critics and concerns regarding the effectiveness of PEMF treatment for fracture healing. One notable criticism is the inconsistency of results, which may be attributed to factors such as the low achievable impulse frequency of most PEMF devices, resulting in a lower biological impact. Practitioners have also expressed concerns about the lengthy treatment sessions required. In contrast, EMTT devices, producing a 40 × higher oscillation frequency, may provide noticeable clinical effects more rapidly. A follow-up study involving 1382 patients with delayed union and non-union fractures who received varying durations of PEMF stimulation showed a median healing time reduction of 35–60% [38]. Patients receiving ten hours of PEMF stimulation per day experienced healing 76 days earlier than those receiving only three hours per day. This study emphasizes the importance of more efficient electromagnetic wave therapy. Since EMTT is a relatively new form of electromagnetic wave therapy, the available evidence on its effectiveness is limited. An in vitro study investigating its influence on human bone marrow mesenchymal stem cells (MSCs) demonstrated increased VEGF concentration and a tendency toward higher expression of bone formation-specific genes (collagen I and alkaline phosphatase). These findings support the concept that electromagnetic waves can play a beneficial role in promoting bone fracture healing through mechanisms involving bone metabolism and angiogenesis [17]. A case report in the literature on EMTT describes a novel approach involving the combination of EMTT with high-energy focused extracorporeal shockwave therapy (ESWT) for treating a non-union of the metacarpal V bone [39]. The patient underwent a specific treatment protocol, including three ESWT sessions followed by EMTT stimulation. This dual therapy was administered once a week for a total of three weeks. Four weeks after the completion of the therapy, there was evidence of enhanced bony consolidation in the metacarpal bone. This case report highlights a potential advanced therapy concept for treating bone-specific disorders. Additional studies are necessary to determine the optimal treatment protocols for different types of bone disorders involving EMTT and ESWT. These studies would help establish the most effective parameters, frequencies, durations, and combinations of these therapies to maximize their therapeutic benefits in various bone-related conditions. A better understanding of the optimal protocol would aid in providing evidence-based guidelines for healthcare professionals and ensure the best possible patient outcomes.

## Conclusion

Initiation of EMTT treatment soon after a bone fracture, regardless of whether the patient undergoes surgery or receives non-surgical treatment, could serve as an essential adjunct therapy to expedite recovery time and facilitate a return to sports activities, whether at an amateur or professional level.

## Data Availability

All data generated or analysed during this study are included in this published article.

## Abbreviations

EMTT: Extracorporeal magneto-transduction therapy
ESWT: Extracorporeal shockwave therapy
AAOS: American Academy of Orthopaedic Surgeons
PEMF: Pulsed electromagnetic field
FDA: Food and Drug Administration
TT (-Bike/-Position): Time trial
NSAIDs: Nonsteroidal anti-inflammatory drugs
MSCs: Mesenchymal stem cells
VEGF: Vascular endothelial growth factor
